# Can laboratory blood tests be used to risk stratify patients admitted with pneumonia?

**DOI:** 10.1186/cc13443

**Published:** 2014-03-17

**Authors:** L Apps, S Hutchinson, T Leary, L Perry

**Affiliations:** 1 Norfolk & Norwich University Hospitals NHS Foundation Trust, Norwich, UK

## Introduction

Our objective was to determine whether an Early Warning Score (EWS) based on laboratory tests could risk stratify patients admitted to the Norfolk & Norwich University Hospitals NHS Foundation Trust with pneumonia. Physiological EWS systems producing an escalated response to an increasing score are used effectively within the Trust, yet mortality is high in patients admitted with a diagnosis of pneumonia. The CURB-65 score may not be an effective risk stratification tool for predicting mortality in the older patient nor to guide intensive care admission [1]. Jarvis and colleagues developed an EWS based on laboratory blood tests and used it in conjunction with physiological EWS to effectively risk stratify all medical patients [2].

## Methods

Of 2,158 patients admitted with pneumonia during 12 months from August 2012, data were collected for 1,598 who had received the required blood tests. Data included dates of admission, discharge and death if appropriate, gender, haemoglobin (g/dl), white cell count (10^9^/l), sodium (mmol/l), potassium (mmol/l), urea (mmol/l), creatinine (mmol/l) and albumin (g/l) on admission. A composite EWS was calculated and measured against outcome.

## Results

Of 1,598 patients, 538 died during this admission. It is uncertain whether death was due to pneumonia as only admission diagnosis is recorded but overall mortality was 35%. Analysis of data showed a strongly positive relationship between increasing EWS and increased risk of mortality with a correlation coefficient of 0.97.

## Conclusion

Observational results suggest an EWS based on laboratory tests can be used to risk stratify patients with pneumonia and could be used to treat those with higher scores more aggressively earlier in their illness. Further analysis is required to determine whether age is a contributing factor and how this may modify the EWS. We need to determine whether laboratory-based EWS risk stratification can be used in isolation, or whether it contributes sufficiently to an existing physiological EWS that a combined system would improve outcome in our patients.
